# Mycobacterium avium subsp. hominissuis effector MAVA5_06970 promotes rapid apoptosis in secondary-infected macrophages during cell-to-cell spread

**DOI:** 10.1080/21505594.2018.1504559

**Published:** 2018-08-23

**Authors:** Lia Danelishvili, Rajoana Rojony, Kylee L. Carson, Amy L. Palmer, Sasha J. Rose, Luiz E. Bermudez

**Affiliations:** a Department of Biomedical Sciences, College of Veterinary Medicine, Oregon State University, Corvallis, OR, USA; b Department of Microbiology, College of Science, Oregon State University, Corvallis, OR, USA

**Keywords:** M. avium, macrophages, MAVA5_06970, MAV_1445, SPP1, osteopontin, apoptosis, IL-12

## Abstract

Mycobacterium avium subsp. hominissuis is an opportunistic intracellular pathogen associated with disease in patients either immunosuppression or chronic lung pathology. Once in the host, M. avium preferentially infects and replicates within the phagocytic cells. The host driven macrophage apoptosis appears to be an essential aspect of innate immunity during bacterial infection; however, the existing evidence suggests that M. avium has evolved adaptive approaches to trigger the phagocyte apoptosis, exit apoptotic cells or via ingestion of infected apoptotic bodies subsequently infect neighboring macrophages. By evaluating 4,000 transposon mutants of M. avium in THP-1 cells, we identified clones that can trigger a new form of early host cell apoptosis, which is only observed upon entry into the “secondary-infected” macrophages. Inactivation of MAVA5_06970 gene lead to significant attenuation in intracellular growth within macrophages and mice, and impaired M. avium to induce rapid apoptosis in the “secondary-infected” cells as measured by Annexin V-FITC detection assay. Complementation of MAVA5_06970 gene corrected the attenuation as well as apoptotic phenotypes. The MAVA5_06970 gene encodes for a secreted protein. Using the pull-down assay and then confirmed with the yeast two-hybrid screen, we found that MAVA5_06970 effector interacts with the Secreted Phosphoprotein 1, the cytokine also known as Osteopontin. This interaction enhances the THP-1 cell apoptosis and, consequently, restricts the production of interleukin-12 that likely may limit the activation of the type I immunity pathway in vivo. This work identified a key virulence effector of M. avium that contributes to the cell-to-cell spread of the pathogen.

## Introduction

It is anticipated that infections by Mycobacterium avium subsp. hominissuis (M. avium) are originated from exposure to environmental sources as M. avium is ubiquitously spread in soil and water, and biofilms of this hardy pathogen can be found in municipal water sources. M. avium typically is an opportunistic pathogen, and infections occur mostly in immunocompromized population such as HIV/AIDS, in patients with chronic lung pathology and in individuals undergoing immunosuppressive therapy [1–3]. Recent data supports that nontuberculous mycobacterial pulmonary infections, including infections by M. avium in individuals without any underlying conditions, are increasing in prevalence across all regions of the United States [4], and lung diseases caused by M. avium complex is currently more common in the US than tuberculosis [5]. It has been also recognized that human activities directly impact on bacterial ecology and selection for M. avium growth, resulting in predominance in human habitats and thereby influencing on epidemiology [6].

M. avium is a successful pathogen that can infect wide range of host cells but predominantly macrophages, and thrives in specialized membrane-bound vacuoles, where bacteria subvert many cellular killing processes. Microbial killing not only depends on the toxic cellular environment but also on the scarcity of nutrients in the phagosomal compartment that M. avium occupies. Despite all, the pathogen actively prevents the vacuole acidification as well as the influx of many toxic compounds into the phagosome by blocking its fusion with late endosomes and lysosomes [7], and hijacks intracellular trafficking pathways to prevent destruction by macrophages [8,9]. M. avium is capable to resist to autophagic killing by phagocytic cells [10] and avoids effects of toxic products such as superoxide anion, nitric oxide, and bactericidal peptides such as cathelicidin and defensins [11–13]. Although macrophage apoptosis is an innate defense mechanism and is a strictly regulated process, M. avium escapes apoptotic killing [8,14]. It has been demonstrated that the pathogen targets intrinsic pathway to promote the apoptotic death in cultured macrophages and in vivo via production of reactive oxygen species, leading to mitochondrial membrane potential loss [15]. Moreover, M. avium uses apoptosis as one of mechanisms to spread from cell-to-cell and for dissemination [8,10]. M. avium infected macrophages undergo apoptotic process few days (3 to 5) after infection, where some bacterial subpopulation either escape from the apoptotic cells to the extracellular space or remain in the apoptotic bodies. In both scenarios, surrounding host macrophages attempt to ingest and eliminate the extracellular bacteria and/or clear the apoptotic bodies. These macrophages subsequently become infected (secondary-infection) with M. avium that has survived the first-line defenses. Recent studies have shown that a host-adapted phenotype of M. avium becomes amplified after the infection of the primary macrophages, allowing bacteria to infect a “second”, uninfected population of macrophages with significantly greater efficiency via complement receptor 3 independent mechanism [16]. Moreover, vacuole environments of primary- and secondary-infected macrophages differ significantly from each other completely changing M. avium behavior [17]. The pathogen leaving the primary macrophages gains more invasive/virulent phenotype and triggers a new, rapid form of the host cell apoptosis, which is only observed upon entry into the secondary-infected macrophages. This predominant phenotype is more commonly associated with the spreading of the bacteria [8,10].

To identify M. avium virulence factors that are associated with induction of the rapid apoptosis of secondary-infected macrophages and, consequently, contributing to the cell-to-cell spread of the pathogen, we evaluated approximately 4,000 transposon mutants of M. avium in THP-1 human cells. This study identified a key virulence gene of M. avium and molecular mechanism by which MAVA5_06970 effector protein enhances apoptotic death and interferes with the activation of the type I immunity pathway in the host macrophages, aiding the pathogen growth within secondary cells and enabling rapid exit from infected phagocytic cells.

## Results

### Initial screening of M. avium mutant library and conformational study

The M. avium A5 transposon bank was partially screened (4,000 mutants) for the lack of ability to induce apoptosis in secondary macrophages after leaving the primary infected cells. Previously, our group has demonstrated that M. avium infected macrophages undergo apoptosis approximately 4 days post-infection [16]. In addition, infected phagocytes that become apoptotic detach from the tissue culture wells [18]. We used this specific characteristic to screen for M. avium gene knockout clones that were incapable of escaping the primary infected phagocytes as the wild type strain in previous study [8] and clones that exit primary macrophages in a similar manner as the wild type but expressed delayed apoptotic phenotype in secondary infected cells (this study). Extracellular fraction of M. avium clones that exit the primary cells at five days post-infection were used to infect secondary macrophages. The initial selection for deficient clones to induce secondary macrophages apoptosis was performed based on the visual screening. While secondary THP-1 monolayers infected with the wild-type M. avium A5 after two days of infection quickly undergo apoptosis, and approximately 20–30% cells detached from monolayers, we could not observe similar occurrence for ten clones listed in the Table 1.

**Table 1.** M. avium A5 genes associated with the rapid apoptosis in secondary-infected human macrophages.

Mutant clone	Accession Gene Description		Homology		
			M. avium 104		M. tuberculosis H37Rv
AM3_28	A0A0E2WBJ9	Intergenic	50bp before MAVA5_13545, pyruvate kinase	MAV_3170	Rv1617
AM4_31	A0A0E2WUW5	MAVA5_03425	TetR transcriptional regulator	MAV_3805	none
Multiple	A0A0E2WCL9	MAVA5_06200	Chaperone DnaJ domain containing protein	MAV_1286	none
Multiple	A0A0E2W9D0	MAVA5_06720	Major Facilitator Superfamily (MFS) transporter	MAV_1387	Rv1250
AM4_32	A0A0E2W8Z5	MAVA5_06970	Hypothetical protein	MAV_1445	none
AM1_18	A0A0E2W504	MAVA5_13300	Adenylate cyclase	MAV_3122	Rv1647
Multiple	A0A0E2W528	MAVA5_18200	Short-chain dehydrogenase	none	none
AM4_30	A0A0E2W2P8	MAVA5_18830	Thiosulfate sulfurtransferase	MAV_4253	Rv3283
Multiple	A0A0E2WK23	MAVA5_20300	Acyltransferase	MAV_4648	Rv0502
AM4_29	A0A0E2VZZ6	MAVA5_21795	AMP-dependent synthetase and ligase	MAV_4950	none

For apoptosis conformational studies in the secondary macrophages, MAVA5_06200, MAVA5_06970, MAVA5_13300 and MAVA5_21795 gene knockout clones that grew in the culture medium in a similar fashion as the wild type bacteria were selected out of ten clones and further evaluated DNA fragmentation by TUNEL assay. As shown in the Table 2, all four clones were found to be deficient to induce quick apoptosis after 48h post-infection of THP-1 cells, and the difference was significant (p < 0.05) between apoptosis rates of secondary-infected THP-1 macrophages of the wild type and all selected gene knockout clones. The wild type M. avium triggered approximately 18–21% greater levels of apoptosis in secondary macrophages when compared with apoptosis deficient knockout mutants at the same time point.

**Table 2.** THP-1 cell apoptosis in response to M. avium secondary infection measured by TUNEL assay.

Clone	% Apoptosis/200 cells in secondary infection
M. avium A5	26.5 ± 7
MAVA5_06200(-)	6 ± 3*
MAVA5_06970(-)	8 ± 4*
MAVA5_13300(-)	6 ± 2*
MAVA5_21795(-)	5 ± 3*

*, p < 0.05 compared with the wild type M. avium A5 strain infection at 48h

### Apoptosis deficient M. avium mutants of secondary macrophages are efficiently cleared in mice

**Figure 1.**
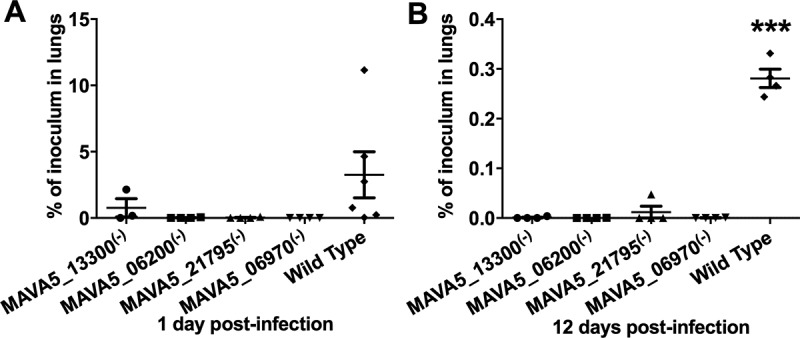
In vivo survival of M. avium A5 wild type and gene knockout mutants expressing delayed apoptotic phenotype in secondary infected macrophages after 24h (A) and 12 days (B) post-infection. ***, p < 0.0001 between the wild type control and all mutant infections at day12.

M. avium A5 gene knockout clones of MAVA5_06200(-), MAVA5_06970(-), MAVA5_13300(-) and MAVA5_21795(-) that were not capable to induce the macrophage apoptosis upon leaving the primary cells were tested in vivo. To establish whether the identified mutants were attenuated in mice, C57BL/6 ten black mice per group were infected by aerosol into the airways. Five mice were harvested after 24h and two weeks post-infection from the control (the wild type) and experimental (gene knockout mutants) groups. The bacterial loads were determined in lungs at each time point. The serially diluted homogenates were plated on to 7H10 agar and the viable bacterial number was recorded as CFU counts. As shown in Figure 1, while bacterial number in the wild type M. avium A5 infected mice increased over time, all four mutants showed significant attenuation in ability to grow in mice.

Ultimately, we selected MAVA5_06970 gene knockout clone to investigate the molecular mechanism associated with the bacterial inability to leave apoptotic macrophages.

### Characterization of MAVA5_06970 gene knockout clone

**Figure 2.**
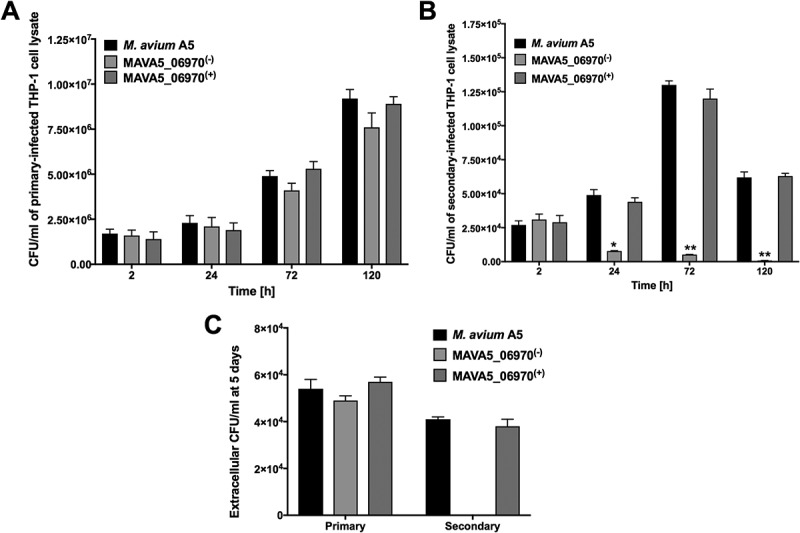
M. avium survival rates in (A) primary and (B) secondary-infected macrophages. THP-1 monolayers were infected with M. avium A5 wild type, the gene knockout mutant MAVA5_06970(-) or complemented MAVA5_06970(+) clone as described in materials and methods. (C) The colony forming units of extracellular bacterial were also evaluated in the supernatants of THP-1 macrophages at 5 days post-infection. *, p < 0.05 and **, p < 0.001 compared with either the wild type M. avium A5 strain or the complemented clone.

Sequencing analysis revealed that transposon insertion at the 7 amino-acid (aa) site of 96 aa protein disrupted translation of MAVA5_06970. The bioinformatic analysis of the MAVA5_06970 protein indicated only a domain of DUFF732 (unknown function) with 3.49E-09 confidence. Using the sequenced-based prediction for secreted proteins and SignalP 4.1, the presence of a 21-aa signal peptide and export via the Sec system were predicted. We next complemented MAVA5_06970 gene in knockout clone, and examined for intracellular growth rate in primary and secondary THP-1 cells (Figure 2). In vitro studies revealed that while the wild type M. avium A5 strain and the MAVA5_06970 complemented clone showed similar survival rates within the primary-infected THP-1 cells, the MAVA5_06970 knockout mutant was slightly attenuated in growth within the primary macrophages when compared to the wild type or complemented group (Figure 2(A)). All clones were capable to leave primary macrophages at a similar rate calculated by CFU counts of extracellular bacteria released in supernatants of M. avium A5, MAVA5_06970 mutant and complemented clone infected THP-1 cells after 5 days post-infection (Figure 2(C)). A significant impact in bacterial growth was observed within secondary-infected macrophages (Figure 2(B)). The intracellular viability, however, was fully recovered in secondary-infected macrophages by complementing the mutant with the functional MAVA5_06970 gene. In addition, it was observed that the inactivation of the MAVA5_06970 gene did not lead to any significant loss of invasion in both macrophages and mutant invaded THP-1 cells but over time it showed significant attenuation in intracellular growth in secondary-infected cells when compared with the wild-type and complemented clone infection (Figure 2(B)). While the extracellular wild type M. avium A5 and complemented clone CFU counts significantly increased in supernatants of secondary-infected macrophages at 5 days post-infection, we could not detect the MAVA5_06970 mutant release in the supernatants of macrophage monolayers at the same time point (Figure 2(C)).

**Figure 3.**
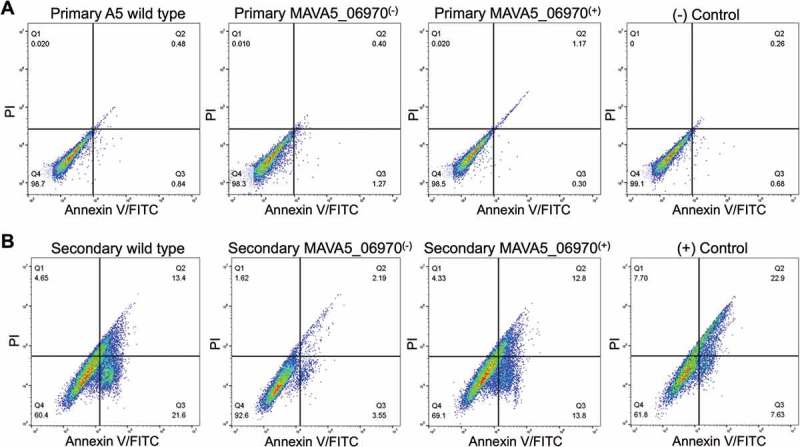
Percent of apoptosis of (A) primary and (B) secondary-infected THP-1 cells following 48h infection with M. avium A5 wild type, the MAVA5_06970(-) mutant and MAVA5_06970(+) clone. The percentage of early and late apoptotic THP-1 cells labeled with the Annexin V/FITC were determined by evaluating fifty thousand cells by flow cytometry in three separate experiments.

Furthermore, the MAVA5_06970 complemented clone was tested for competency to restore the pro-apoptotic phenotype using the Annexin V staining assay. The primary and secondary THP-1 macrophages were infected with the wild type, mutant and complemented clone for 48h and then processed for the Annexin V-FITC staining as described in material and methods. The cell cytotoxicity was quantified using BD accuri C6 flow cytometer (Figure 3). Results demonstrate that while the cell death in 48 hour infected primary macrophages were very similar in all tested groups and caused roughly 1% of the cell death (Figure 3(A)), the secondary infection resulted in much higher percentage of apoptosis at the same time point and showed significant changes between control and experimental groups (Figure 3(B)). The wild type infection of secondary THP-1 cells lead to 35% of death and complemented clone restored the pro-apoptotic fitness to 27%, whereas mutant infection significantly failed to induce cell death and resulted in apoptosis in only 6% of infected cells (Figure 3(B)). The staining of uninfected cells served as a background control and staurosporine treatment as a positive control.

### The MAVA5_06970 gene is predominantly expressed during M. avium early exposure to primary and secondary human macrophages

**Figure 4.**
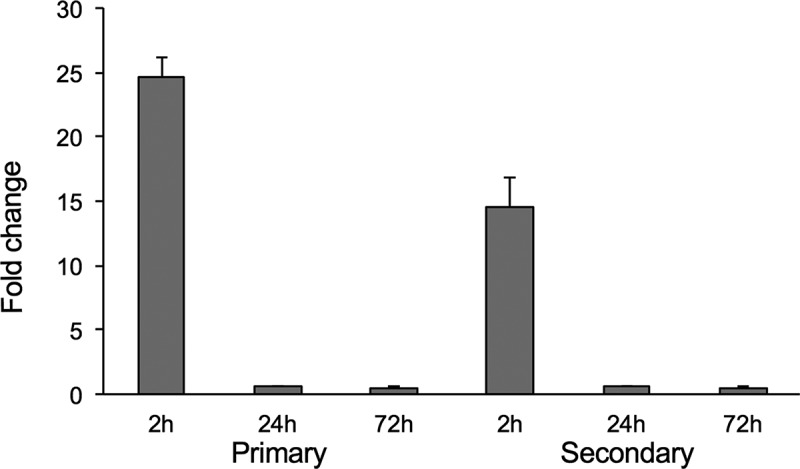
M. avium MAVA5_06970 gene expression following exposure or infection of primary and secondary macrophages. Total RNAs from broth-grown bacteria, as well as from macrophage-exposed and intracellular bacteria, were used to determine the copy numbers of cDNAs for target and reference genes. Quantitation of the expression of the MAVA5_06970 gene was carried out with SYBR Green I assay by Real-Time PCR detection system using gene-specific primers. Results were analyzed on Ct values basis for each sample and normalized with an internal housekeeping gene control 16S rRNA. Data represents mean and standard deviation values from two independent experiments.

To determine the timeline of MAVA5_06970 gene expression, the wild type M. avium A5 was either exposed to THP-1 cells for 2h or infected for 24h and 3 days. At each time points bacterial RNA was isolated from extracellular or intracellular bacteria and then quantitatively analyzed using the Real-Time PCR. The expression levels of MAVA5_06970 were normalized to the expression level of the endogenous reference 16S rRNA for control (bacteria grown in the 7H9 culture medium) and macrophage exposed or intracellular bacterial samples. As shown in the Figure 4, similar trends of MAVA5_06970 gene expression levels were observed in primary and secondary exposed or infected cells; however, there were significant expression levels of MAVA5_06970 gene when M. avium was exposed to primary as well as secondary THP-1 cells. The MAVA5_06970 gene expression of 25-fold was recorded during M. avium initial interaction with primary cells, whereas an exposure to secondary cells resulted to16-fold upregulation when compared to the control. No expression and significant changes were detected in the MAVA5_06970 gene expression for intracellular bacteria at 24h and 72h post-infection of both primary and secondary macrophages (Figure 4).

### Effector MAVA5_06970 protein interacts with the Secreted Phosphoprotein 1 (SPP1), the cytokine also know as Osteopontin

Due to the presence of signal peptide of Sec secretion system in MAVA5_06970 protein as well as significant homology to uncharacterized secreted effectors of M. marinum MMAR_2929 and M. avium subsp. paratuberculosis MAP_2476, we hypothesized that MAVA5_06970 undergoes proteolytic cleavage before secretion, and either it is located on the outer membrane of M. avium or translocated into the cytosol of the host cell and, thus, being capable of interacting with host proteins.

**Figure 5.**
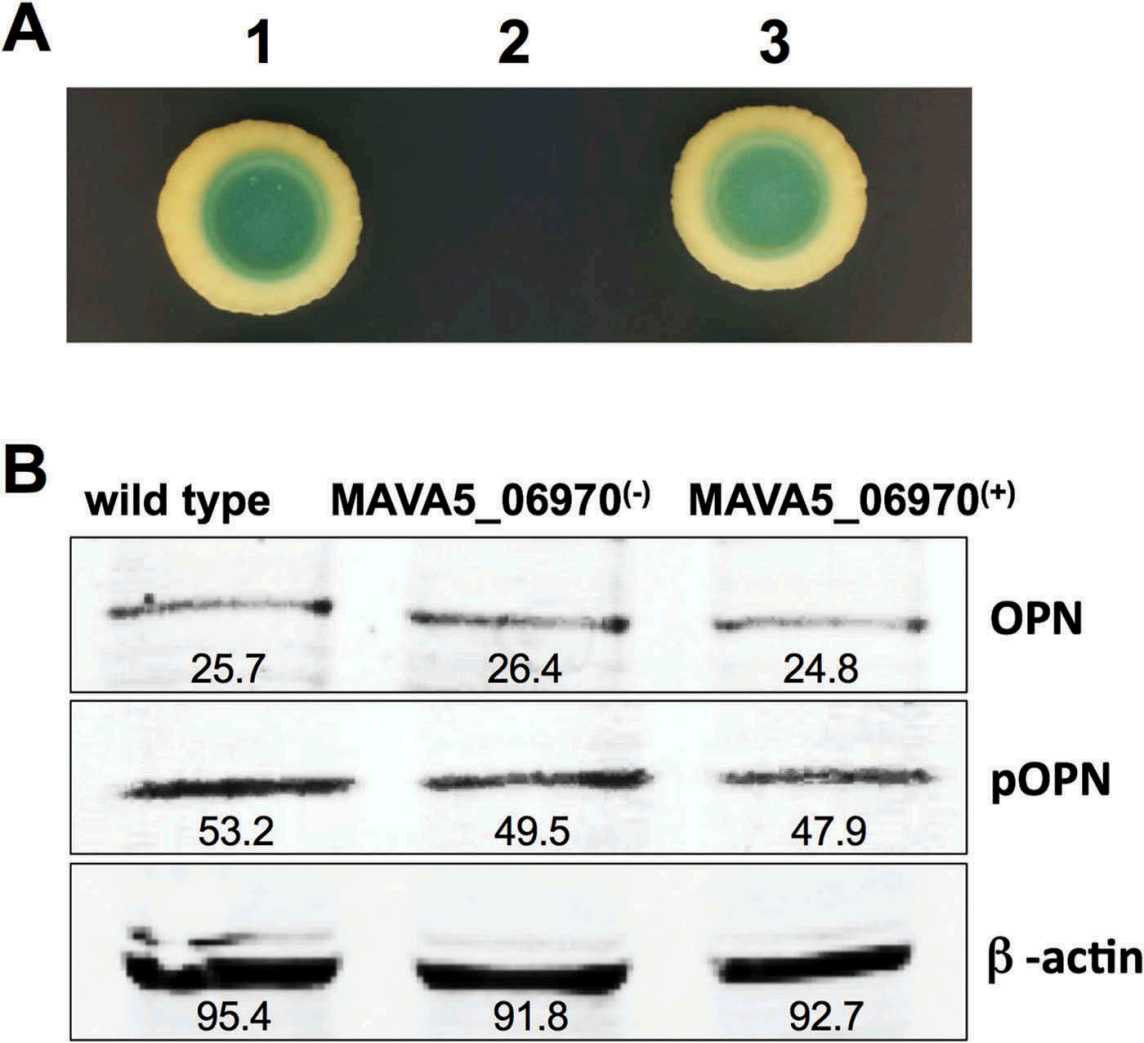
(A) The yeast two-hybrid interaction of MAVA5_06970 with the host target SPP1 protein. The open reading frame of SPP1 encoding a 301 amino acid protein was amplified from the cDNA of THP-1 cells amplified from the total RNA. The yeast two-hybrid screening of MAVA5_06970 established positive interaction with SPP1 (1). The known interaction between pGBKT7-lam and pGADT7-T served as a positive control (3), and pGBKT7-53 and pGADT7-T as a control for a negative interaction (2). (B) OPN levels of secondary THP-1 cells infected with the wild type M. avium, the gene knockout MAVA5_06970(-) mutant and complemented MAVA5_06970(+) clone. After 2h of infection cell lysates were subjected to OPN immunoprecipitation under denaturing conditions by using an agarose-conjugated primary antibody and analyzed via anti-OPN antibody (a) or phosphoserine/threonine/tyrosine antibody (b). The photon emission means were recorded for each band to quantify the signal intensity on the Odyssey Imager (Li-Cor). The β–actin served as a loading control (c).

To investigate potential host binding partner(s) for MAVA5_06970 proteins, pull-down assay was performed with THP-1 cleared lysate. The recombinant MAVA5_06970 protein was produced in E. coli using the pET Expression system and purified with the His-Bind resin chromatography (Clontech) as per the manufacturer’s instructions. The pull-down assay with MAVA5_06970 displayed binding with five host proteins (Table 3). The most noteworthy is the Secreted Phosphoprotein 1. In the conformational study, MAVA5_06970 interaction with SPP1 host protein was tested using the yeast two-hybrid system. The MAVA5_06970 effector demonstrated the positive interaction with Osteopontin cytokine as the resulting yeast zygotes of both the bait and pray constructs grow in the absence of Ade, His, Leu, and Ttp and presence of 125 ng/ml Aureobasidin and X-a-Gal, and turned blue in the presence of X-a-Gal meaning that transcription of all four AUR1-C, ADE2, HIS3 and MEL1 reporters controlled by the Gal4 promoter took place (Figure 5(A)).

**Table 3.** Host proteins interacting with M. avium recombinant MAVA5_06970.

Accession	Description	% Protein Coverage	# Peptides	MW [kDa]
B7Z351	Secreted phosphoprotein 1 variant 6	37.2	16	37.2
P14866	Heterogeneous nuclear ribonucleoprotein L	13.1	3	64.1
E9KL48	Epididymis tissue sperm binding protein Li 18mP	9.9	4	61.4
Q59FF0	EBNA-2 co-activator variant (Fragment)	10.0	7	107.4
P01023	Alpha-2-macroglobulin	7.1	2	163.2

To examine if the MAVA5_06970 interaction with OPN leads to modification of the host protein including phosphorylation, we performed the Western blotting on immunoprecipitated samples using anti-OPN and anti-phosphoserine/threonine/tyrosine antibodies (Figure 5(B)). We did not observe any cleavage or changes in the molecular mass of OPN. There were no apparent modifications in phosphorylation levels of OPN as well in all tested groups.

### The MAVA5_06970 knockout clone fails to inhibit IL-12 secretion in THP-1 macrophages

The SPP1 protein (same as Osteopontin) acts as a cytokine involved in activation of the type I immunity through enhancing the production of interleukin-12, interferon-gamma and reducing production of interleukin-10. It has been established that M. avium infection initially triggers IL-12 synthesis by phagocytic cells but overtime with the progression of the infection it is capable to suppress the IL-12 secretion. We decided to test this phenotype using the MAVA5_06970 mutant. The human macrophage monolayers were infected with M. avium A5, the MAVA5_06970 knockout mutant and complemented clone, and IL-12 production in the supernatants was measured after 24, 48, and 72 h post-infection. As shown in Table 4, both the wild type and complemented clone of M. avium A5 were capable to induce IL-12 production at all time points in primary-infected THP-1 cells, however, a significant suppression of IL-12 synthesis was observed in 48 h and 72 h infected monolayers, and inhibition was even more remarkable in secondary-infected cells showing no detectable levels at 72h post-infection (Table 4). While similar levels of IL-12 secretion was observed in MAVA5_06970 mutant infection at 24h when compared to the wild type and complemented clone infection in primary cells, no decrease in IL-12 production was seen at 48h and 72h (Table 4). This observation was true for secondary infected macrophages as well. IL-12 production was not detected in supernatants of uninfected macrophages (control).

**Table 4.** IL-12 synthesis by primary- and secondary-infected THP-1 cells.

Clonea	IL-12b (pg/ml) Primary Secondary					
	24h	48h	72h	24h	48h	72h
M. avium A5	87 ± 5	43 ± 4	18 ± 7	48 ± 6	19 ± 8	Undetectable
MAVA5_06970(-)	69 ± 6	83 ± 7	98 ± 5	75 ± 12	80 ± 5	93 ± 4
MAVA5_06970(+)	73 ± 12	36 ± 8	20 ± 3	38 ± 9	20 ± 4	Undetectable
Uninfected control	Undetected	Undetected	Undetected	-	-	-

^a^The macrophage monolayers were infected with a multiplicity of infection 10 bacteria: 1 cell. *P < 0.05 compared with the level of IL-12 in supernatant of 24 h infected cells. bIL-12 (p40/p70) minimum detectable limit is < 2 pg/ml.

### M. avium MAVA5_06970(-) mutant infection of THP-1 cells in combination with the recombinant MAVA5_06970 protein restores apoptotic phenotype and inhibits IL-12 secretion

**Figure 6.**
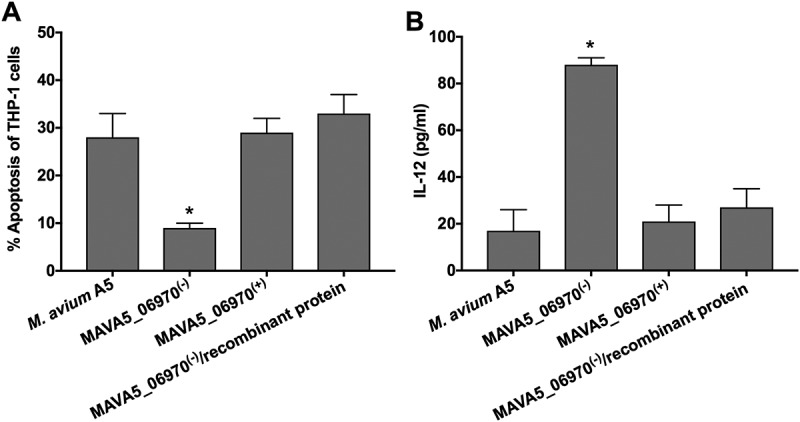
The recombinant MAVA5_06970 protein restores the MAVA5_06970(-) mutant phenotype in secondary-infected macrophages. (A) THP-1 macrophage monolayers were seeded in 8-chammber glass slides and infected either with the wild-type, MAVA5_06970 gene knockout mutant, complemented clone or MAVA5_06970(-) exposed to 100μg/ml recombinant MAVA5_06970 protein. Infection was carried out for 2h with the MOI of 10:1, and then monolayers were washed 3 times with PBS. After 48h, apoptosis were analyzed using the TUNEL assay. The percentage of apoptosis was calculated in 200 secondary-infected THP-1 cells. *, p < 0.05 compared with the wild type M. avium A5, complemented and MAVA5_06970(-) infection in combination with the MAVA5_06970 recombinant protein at 48h post-infection. (B) THP-1 macrophage monolayers seeded in 24-well plate were infected/exposed to recombinant protein as describe above and supernatants were collected at 48h post-infection for IL-12 measurement using ELISA assay. *P < 0.05 between mutant and the wild type, complemented and mutant infection in combination with the MAVA5_06970 recombinant protein at 48h post-infection. Data represents mean and standard deviation values from two independent experiments.

The MAVA5_06970 gene encodes a secreted protein, and it is highly expressed within 2h infection of macrophages suggesting MAVA5_06970 secretion and, most likely, binding to OPN cytokine during invasion of THP-1 cells. To test our hypothesis, we used the recombinant MAVA5_06970 protein during MAVA5_06970(-) mutant infection and apoptotic phenotype in secondary-infected macrophages as well as IL-12 secretion were evaluated together with the wild type and complemented strain infection (Figure 6). As shown in the Figure 6(A), exposure of the MAVA5_06970 gene deficient mutant with the recombinant protein during 2h infection of THP-1 cells significantly changed the outcome and induced quick apoptosis in secondary-infected macrophages evaluated by TUNEL assay at 48h post-infection. The apoptosis rates were found to be similar to the one observed with the wild type and complemented clone (Figure 6(A)). In addition, evaluation of IL-12 levels in the wild type, MAVA5_06970(-), MAVA5_06970(+) and MAVA5_06970(-) with combination of recombinant protein also confirmed that the recombinant MAVA5_06970 restores the mutant’s defect to inhibit the secretion of IL-12 cytokine (Figure 6(B)).

## Discussion

Once M. avium gains entry to the host through either the respiratory or gastrointestinal tract, it is preferentially phagocytized by tissue or blood mononuclear phagocytes. The pathogen spreads from originally infected macrophages to surrounding uninfected phagocytic cells, and then ultimately transported to the local lymph nodes. There is a very limited knowledge on understanding of the molecular basis of M. avium transmission from cell-to-cell. Recent studies have shown that an invasive phenotype of mycobacterium develops after the infection of the primary macrophages, allowing bacteria to leave originally-infected cells and then invading a second, uninfected population of macrophages with significantly greater efficiency [19].

As innate immunity process to control and suppress the infection, macrophages undergo program cell death. In recent years, however, it has been accepted that apoptotic process is emerging as a major trait of bacterial virulence benefiting intracellular pathogens to establish and spread infection [20]. Yersinia translocates type III secretion YopJ/P pro-apoptotic factors that inhibit the activation of the nuclear factor NFkB thereby preventing synthesis of TNFα inflammatory cytokine, eliminating phagocytic cells by apoptosis and promoting bacterial dissemination [21–23]. Studies have shown Salmonella typhimurium to promote apoptosis as an escape mechanism from phagocytic cells by either inducing immediate cell death via caspase-1-dependent pathway using bacterial factors of the pathogenicity island SPI1, or delayed macrophage death after establishing an intracellular niche within vacuoles via SPI2-encoded products [24]. Virulent M. tuberculosis blocks the extrinsic pathway of apoptosis through secreting effector proteins Rv3654c and Rv3655c [18] and interfering with caspases’ post-transcriptional events [18]. While M. tuberculosis inhibits the extrinsic pathway of apoptosis during early stage of infection, later it also activates the intrinsic pathway, leading to the mitochondrial transmembrane damage and macrophage necrosis [18]. Using this strategy dependent on the RD1and other factors, the pathogen undoubtedly promotes bacterial cell-to-cell spread [25,26].

M. avium escapes macrophage killing by expressing MAV2054 virulence factor and promoting the intrinsic apoptotic pathway in cultured macrophages as well as in vivo via production of reactive oxygen species, damaging mitochondrial membrane [15]. Recent studies by our group have demonstrated that M. avium infection stimulates different modes of apoptotic escape in the primary- and secondary-infected cells [10,16]. At first, after uptake by primary phagocytic cells, M. avium can escape apoptotic death by two distinctive mechanisms [8]. Typically, the pathogen remains in phagosome until macrophage apoptosis is triggered and vacuole rupture occurs roughly four days later of infection [10]. Once bacteria is released in the cytoplasm, it is capable to directly exit apoptotic macrophages or remain within apoptotic bodies, resist killing by efferocytosis process and subsequently result to transmission of the pathogen to new subset of macrophages without exposing bacteria to the extracellular space. This, however, is not the case for M. tuberculosis infection, and efferocytosis leads to the pathogen destruction [27]. Few M. avium factors associated with the pathogen escape to extracellular space from apoptotic cells have been identified and have shown to interfere with protein degradation, post-transcriptional regulation and possibly involved in RNA splicing, altering de novo protein synthesis process by macrophages [8].

Recently, we have described a new, rapid form of macrophage apoptosis induced by M. avium, which is only observed upon entry into the “secondary-infected host cells” [8]. Out of ten gene-knockout clones we focused on four that displayed significant attenuation in intracellular growth in cultured macrophages and did not show growth defect in vitro. All four mutants also demonstrated significantly lower percentage of viable bacterial loads in lungs of mice when compared with the wild type infection at one and twelve days post-infection. To further characterize and understand some of the molecular strategies associated with M. avium rapid exit from secondary apoptotic macrophages, we selected the hypothetical MAVA5_06970 containing established signal sequence of Sec secretion system. The bioinformatics analysis has identified highly homologous secreted effector of M. marinum MMAR_2929 proven to undergo to proteolytic cleavage and secreted in culture filtrates [28,29]. We identified an M. avium transposon clone with a nonfunctional MAVA5_06970 gene to be deficient in the induction of rapid macrophage apoptosis and observed that MAVA5_06970 is required for M. avium virulence and survival in macrophages and in mice. Furthermore, protein-protein binding findings confirmed by the Yeast Two-Hyrid interaction study identified the secreted phosphoprotein 1 or commonly known as Osteopontin (OPN) as an interacting partner for MAVA5_06970. Osteopontin, an extracellular matrix protein and proinflammatory cytokine, is implicated in diverse physiological and pathological processes [30], and is expressed in various cells including fibroblasts, endothelial cells, dendritic cells, macrophages, T-cells and natural killer cells [31–33]. OPN plays a critical role during chronic inflammatory diseases including tuberculosis and non-tuberculous infections, and its deficiency predisposes to bacterial dissemination and more severe disease [34]. In fact, direct correlation of OPN expression levels and disease progression and severity has been revealed in patients with M. intracellulare infection, highlighting OPN significance in human resistance against this pathogen [34]. In addition, measuring OPN responses has been proposed to be reliable prognostic indicator for improvement of pulmonary tuberculosis during early phase of treatment [35] and useful tool for testing BCG vaccine safety in vitro before its administration in individuals with underlying immunodeficiencies such as with IFN-γ signaling defects [34].

In this study, we demonstrate that MAVA5_06970 gene is mainly expressed during early interaction with primary as well as secondary phagocytic cells but not throughout the intracellular growth. We do not have full understanding why the expression of MAVA5_06970 gene does not trigger the rapid macrophage apoptosis in primary cells. Perhaps, due to the fact that there are several M. avium pro-apoptotic genes, validated in this study, are involved in initiation of early apoptotic phenotype of secondary macrophages, they need to function together with MAVA5_06970 and concurrent expression of these genes are required, which most likely is the case when M. avium spreads to secondary cells.

A genetic inactivation of the MAVA5_06970 gene leads in attenuation of invasive/virulence phenotype, which normally is activated in the wild-type M. avium after encountering the intracellular milieu of primary macrophages. Furthermore, MAVA5_06970 deficient mutant is unable to promote the rapid apoptotic death during dissemination to secondary/uninfected macrophages; the trait that helps M. avium to efficiently spread from-cell-to-cell. It has been established that the MAVA5_06970 interacting partner Osteopontin is involved in monocyte differentiation to macrophages [36], immune cell activation, attachment and migration [37], and inhibition of autophagy by stimulating the p38 MAPK signaling pathway [38]. OPN can inhibit not only the host macrophage apoptosis by interacting with α4 integrin and CD44 [39] but is capable to prevent the apoptotic death of endothelial cells, melanocytes, renal epithelial cells, and IL-3-dependent hematopoietic cells as well [37,40–42]. To examine if the MAVA5_06970 interaction with OPN resulted in any modification the host protein or altered phosphorylation levels, we performed the Western blot analysis of OPN on whole-cell lysates of THP-1 macrophages infected with the wild type M. avium, mutant, or a complemented clone. We did not observe any cleavage or changes in the molecular mass of OPN as well as no altered phosphorylation patterns were detected, suggesting that MAVA5_06970 binding to OPN mainly interfered the OPN function by making the host protein non-functional.

OPN has a pivotal role to facilitate the recruitment of phagocytic cells and promote the cytokine secretion, in particular, shaping up the immune response of phagocytic cells by inducing production of IL-12 and inhibiting IL-10 [39]. The fact is that endogenous IL-12 is necessary for induction of effective protective immunity against M. avium as well as M. tuberculosis [43,44]. Recent studies suggest that M. avium infection of macrophages results in progressive decrease of IL-12 production by infected cells and in mice [45]. Next, we asked whether the interaction of MAVA5_06970 with OPN had a downstream effect on IL12 production in human macrophages. Indeed, we found that while diminished levels in IL-12 production was obvious overtime in the wild type M. avium and complemented clone infected primary and secondary cells, the MAVA5_06970 clone failed to inhibit IL-12 synthesis, and correlated to decreased number of viable bacteria.

In summary, we characterized the MAVA5_06970 virulence effector that is required for M. avium survival and cell-to-cell spread via apoptosis. The underlying mechanism of the MAVA5_06970-OPN interaction as a cause of apoptosis remains unclear; however, an indirect mechanism cannot be excluded at this time. Based on our work, we can conclude that M. avium limits OPN anti-apoptotic and pro-inflammatory function via MAVA5_06970, and this process most likely further encourages the pro-apoptotic activity of other bacterial factors. This finding provides new insight into the mechanism on how M. avium adapts and manipulates the host defensive responses to promote bacterial dissemination and pathogenesis in the host.

## Materials and methods

### Bacteria and growth conditions

M. avium clinical strain A5 isolated from the blood of an AIDS patient was used for the described assays. M. avium A5 was grown at 37°C with vigorous shaking for 7–10 days in Middlebrook 7H9 broth supplemented with oleic acid, albumin, dextrose, and catalase, OADC (Hardy Diagnostics). M. avium A5 transposon library was created as previously reported [46]. The transposon MAVA5_06970 (homologues to MAV_1445 of M. avium 104) gene knockout clone was grown in presence of 400 μg/ml of kanamycin, harvested and used in assays.

### Mycobacterial infection assays

An M. avium A5 transposon library of 4,000 clones was screened for decreased levels of apoptosis in comparison to the wild-type bacterium at two days post-infection of secondary-infected THP-1 monocytic cell line. Briefly, the primary THP-1 cells were grown in RPMI-1640 supplemented with 10% fetal bovine serum (Sigma), treated with 20 ng/ml Phorbol 12-Myristate 13-Acetate (PMA, Sigma), and seeded at 1 × 105 mononuclear cells/well in 96-well flat-bottomed tissue culture plates. The primary monolayers were infected with transposon mutants and the wild type at a ratio of 10 bacteria∶1 cell and incubated for 2 h at 37°C and 5% CO2. The wells were washed three times with HBSS to remove extracellular bacteria and incubated for additional 5 days at 37°C. After that time, extracellular bacteria were recovered from supernatants of primary infected cells and used for infection of secondary macrophages. For initial screening, individual wells were visually screened for detached monolayers compare with the control. M. avium clones were selected from wells that had most cells attached and significantly differed from the wild type control infection. Identified clones were sequenced by Ligation-Mediated PCR (LM-PCR) as previously described [46] and further evaluated for invasion and survival assays in primary versus secondary THP-1 macrophages by bacterial colony forming units (CFUs).

### Complementation of MAVA5_06970 gene knockout transposon clone

The 288-bp coding fragment of MAVA5_06970 was PCR-generated from M. avium A5 DNA and cloned into the mycobacterium chromosomal integration vector pMV306 containing the apramicyn resistance marker. The resulting vector was electroporated into the transposon clone, and M. avium transformants were plated on 7H10 Middlebrook agar plates containing 400 µg/ml of apramycin. The positive complemented clone was identified by PCR using MAVA5_06970 gene specific primers. The MAVA5_06970 knockout clone served as a negative control.

### Invasion and survival assays

THP-1 macrophages grown in RPMI-1640 supplemented with 10% fetal bovine serum (Sigma) were differentiated with 20 ng/ml PMA (Sigma) into 12-well plates for primary infection and into 48-well plates for secondary infection. Bacteria recovered from primary cells at day 5 were used for secondary macrophage infection studies. The THP-1 cell monolayers were infected with MOI of 10:1 for 2h. Dilutions of bacterial inoculum were plated to calculate the original concentration of inoculum and invasion percentage in both primary and secondary infected cells at 2h of post-infection. At this point, monolayers were washed three times with HBSS and treated with amikacin 200µg/ml for 1h to kill any reminder extracellular bacteria. The number of phagocytosed bacteria was determined by lysing the monolayer with 0.1% triton X100. The obtained lysate was serially diluted and plated onto 7H10 agar plates. The infection was carried out up to 5 days and dilutions of primary- and secondary-infected cell lysates were plated at 24h, day 1, 3 and 5 post-infection. In addition, extracellular bacterial number was quantified in supernatants of infected macrophages at the same time points. The CFU/ml of cell lysate and the supernatant of the wild type M. avium A5 strain were compared to the MAVA5_06970 knockout and complemented clone infections.

### Mice infection

C57BL/6 black mice (10 mice/group) were infected via intravenous either with the wild-type M. avium A5 strain or the transposon mutant as previously described [47]. After 24h post-infection, five mice were harvested from the control and experimental groups to determine the initial tissue load (lung). The other five mice were harvested at 12 days of infection. Lungs were obtained and homogenized as previously described [47]. The serially diluted homogenates were plated on 7H10 agar supplemented with carbenicillin, amphotericin B, trimethoprim-sulfametoxazol, and polymyxin B, and incubated for 20 days to determine bacterial CFUs. The differences in bacterial CFU per gram of tissue were compared between control and experimental groups at each time point. This study was approved by the IACUC under the protocol number 4396.

### Apoptosis assays

The primary and secondary THP-1 macrophage monolayers seeded in either 8-chammber glass slides or 24-well plates and infected with the wild-type, selected MAVA5_06970 gene knockout and complemented clone with MOI of 10:1 as described above, and then processed for apoptosis assays using the TUNEL (Abcam) and Annexin V FITC assay kits (Cayman). For TUNEL assay, infected cells were permeabilized with detergent solution of 0.1% Triton X100 in PBS and 4% FBS for 5 min at room temperature, washed twice with PBS and incubated for 1h at 37C with 50 ul DNA labeling solution consisting of TdT reaction buffer, TdT enzyme, Br-dUTP and double distilled water as per manufactures instructions. Cells were exposed to 100μL of anti-BrdU-Red antibody in rinse buffer and immediately processed for analysis by the fluorescent microscopy.

For the Annexin V-FITC apoptosis assay, primary and secondary THP-1 cell monolayers were infected with either M. avium A5, the MAVA5_06970 knockout mutant or complemented clone for two days, and then according the manufacturer’s protocol (Cayman) incubated with the binding buffer containing the cell-based Annexin V FITC and propidium iodide for one hour at 4°C. Live cells were detached using TrypLE (ThermoFisher), washed with PBS, fixed in 2% paraformaldehyde solution and processed for the flow cytometry as described elsewhere [48].

### RNA source, total RNA extraction and Real-Time PCR quantification

The extracellular wild type M. avium A5 exposed to primary and secondary THP-1 cells for 2h and intracellular M. avium isolated from infected primary and secondary THP-1 macrophages at 24h and 72h were used as sources for total bacterial RNA isolation. RNAs extracted from M. avium grown till exponential phase on 7H10 Middlebrook agar plate and then exposed to 7H9 broth for 2h, 24h and 72h served as controls. THP-1 macrophage monolayers of 75cm2 flasks were lysed with 0.1% Titon X100 and centrifuged at 300× rpm for 5 min to remove lysed cells from the suspension. After, the supernatant was centrifuged at 3,500× rpm for 10 min and the pellet was used for bacterial RNA extraction. Total bacterial RNA extraction was based on the combination of a guanidine thiocyanate-based buffer (Trisol) (Invitrogen) and rapid mechanical cell lysis in a bead-beater, as previously described [19]. RNA was cleaned up with RNA clean kit (QIAGEN) and treated with DNase I (ThermoFisher Scientific), and RNA concentrations were verified by OD260/280 nm absorption. The first-strand cDNA was synthesized using the iScript cDNA synthesis kit (BioRad) according to the manufacturer’s instructions. Quantitation of the MAVA5_06970 gene expression was carried out in the iCycler iQ (BioRad) with SYBR Green I assay and the gene specific TGAAGGAACTGTTCAGGCCG forward and GGGTACCTCGCCCAATTGAA reverse primers. The calculated threshold cycle (Ct) was normalized to the Ct of the internal control 16S RNA gene amplified from the corresponding samples. The fold change in the gene expression was calculated as previously described [19].

### Preparation of recombinant MAVA5_06970 and pull-down assay

The MAVA5_06970 gene without signal sequence was amplified form the M. avium A5 chromosomal DNA with sense TTTAAGCTTTTATGCATTCCGGCC and antisense TTTAAGCTTGGGGCAGTAGTTGGA primers and cloned into HindIII site of the pET6xHN-N vector (Clontech). The resulting construct was sequenced to confirm the right orientation and fusion with 6xHN-tag. The protein was expressed in E. coli strain BL21 (DE3) according to the manufacturer’s instruction (Clontech). E. coli with induced MAVA5_06970 and the control clone containing pET6xHN-N vector were lysed by mechanical disturbed in bead-beater, and cleared via centrifugation and filtration through 0.2 m filter. Alternatively, THP-1 macrophages were harvested from the 75mc2 flask, washed and mechanical lysed in PBS supplemented with the protease inhibitor cocktail (sigma). Cell lysate was cleared by centrifugation at 500 rpm for 10 min followed by filtration through 0.2 μm filter. The total protein extract from control or MAVA5_06970 expressed E. coli were loaded to the resin of His60 nickel columns and washed per manufacturers protocol. Prior to the elution step, the total protein extract from THP-1 cells were loaded with the immobilized MAVA5_06970 on the nickel column for overnight at 4°C with rotation. Following day, samples were washed according to the manufacturer’s protocol (Clontech) to remove unbound host cell proteins and eluted. The concentrated elutes were mixed with an equal volume of 2X Laemmli sample buffer (Bio-Rad), resolved on a 12% Mini-Protean precast SDS-PAGE protein gel (Bio-Rad) and coomassie stained. The proteins of interest were excised and processes for In-Gel digestion. Proteins were reduced and alkylated via incubation with equal amounts of DTT and iodoacetamide at a final concentration of 10 mM, and then trypsin digested in solution at 37°C for 5h. The mass spectrometric sequencing by electrospray ionization mass spectrometry (ESI-MS/MS) was performed at the Oregon State University Mass Spectrometry facility.

### The yeast two-hybrid system

The MAVA5_06970 gene was fused in frame with the GAL4 DNA binding domain by inserting the PCR-generated fragment into the EcoRI and BamHI sites of pGBKT7. The resultant bait vector pGBKT7: MAVA5_06970 was transformed into Saccharomyces cerevisiae strain Y2HGold using Yeastmaker Yeast Transformation System 2, according to the manufacturer’s instructions (Clontech). The total RNA from THP-1 cells was isolated and cDNA was synthesized as previously described [49]. The SPP1was generated with gene specific primers and cloned into ClaI and BamHI sites of pGADT7 resulting the fusion with the GAL4 activation domain. The resultant prey vector pGADT7:SPP1 was transformed into the yeast strain Y187 (Clontech). Y187 yeast strain and Y2H Gold yeast strain were grown into the SD/–Leu and SD/–Trp on agar media, respectively. Plasmids pGBKT7-53, pGBKT7-lam, and pGADT7-T obtained from Clontech were used as positive and negative controls for interaction studies. One ml of bait strain was combined with the one ml of prey strain and grown in 2xYPDA liquid medium containing 50 μg/ml kanamycin at 30°C for 24 h. The activation of AUR1-C, ADE2, HIS3, and MEL1 reporters controlled by the Gal4 promoter were screened by plating yeast zygotes on Double Dropout (SD-Leu/–Trp), Triple Dropout (SD – His/–Leu/–Trp), Quadruple Dropout (SD–Ade/–His/–Leu/–Trp) and Quadruple Dropout agar plates containing 20 mg/ml X-a-Galactosidase and 125 ng/ml Aureobasidin A (QDO/X/A). AUR1-C confers resistance to Aureobasidin A, ADE2 is required for adenine biosynthesis whereas HIS3 for histidine biosynthesis, and MEL1 produces the enzyme α-galactosidase which can be detected by chromogenic substrate X-α-gal. The colonies that grew of blue color on QDO/X/A were identified as positive clone.

### Western blotting

The THP-1 secondary-infected cells with the wild type M. avium, the gene knockout MAVA5_06970(-) mutant and complemented MAVA5_06970(+) clone were lysed after 2h infection using CelLytic M lysis buffer supplemented with protease inhibitor cocktail (Sigma). Lysates were precleared by centrifugation at 10,000 × g for 15 min to remove the bacterial pellet and cell debris, and then subjected to immunoprecipitation using agarose-conjugated primary OPN antibody (Santa Cruz Biotechnologies, Inc). Samples were separated on 12% Tris-HCl gels and transferred to nitrocellulose. Membranes were blocked with 3% bovine serum albumin (BSA) in PBS containing 0.1% Tween for 1 h and then incubated with either anti-OPN or anti-phosphoserine/threonine/tyrosine primary antibody at a 1:250 dilution for 3 h. Next, membranes were probed with the corresponding IRDye secondary antibody (Li-Cor Biosciences, Inc.) at a dilution of 1:5,000 for 30 min, and proteins were detected using an Odyssey Imager (Li-Cor).

### IL-12 assay

Supernatants of primary and secondary THP-1 cells infected the wild type strain, MAVA5_06970 gene knockout and complemented clones of M. avium as well as supernatants of uninfected cells were collected at 24h, 48h and 72h time points, passed through a 0.22 µ filter and immediately stored at − 80°C. The enzyme-linked immunosorbent assay (ELISA) for quantitative detection of human IL-12p70 were performed and analyzed as per manufacturer’s instructions (Invitrogen). The IL-12 cytokine concentrations were quantified from standard curves obtained from sequential dilutions of the recombinant cytokine.

### Statistical analysis

In vitro experiments were repeated at least three times and the comparison between experimental groups were analyzed using the Prism software (GraphPad) using the Student’s t-test. In addition One-Way ANOVA was used to calculate the results of the mice study.
